# The performance of balance exercises during daily tooth brushing is not sufficient to improve balance and muscle strength in healthy older adults

**DOI:** 10.1186/s12877-021-02206-w

**Published:** 2021-04-17

**Authors:** Urs Granacher, Thomas Muehlbauer, Gerd Göstemeyer, Stefanie Gruber, Markus Gruber

**Affiliations:** 1 Division of Training and Movement Sciences, Research Focus Cognition Sciences, University of Potsdam, Am Neuen Palais 10, Bldg 12, 14469 Potsdam, Germany; 2 Division of Movement and Training Sciences, Biomechanics of Sport, University of Duisburg- Essen, 45141 Essen, Germany; 3 Department of Operative and Preventive Dentistry, Charité - Universitätsmedizin Berlin, Berlin, Germany; 4 Human Performance Research Centre, Department of Sport Science, University of Konstanz, Konstanz, Germany

**Keywords:** Balance, Daily life, Exercise, Healthy aging, Mobility

## Abstract

**Background:**

High prevalence rates have been reported for physical inactivity, mobility limitations, and falls in older adults. Home-based exercise might be an adequate means to increase physical activity by improving health- (i.e., muscle strength) and skill-related components of physical fitness (i.e., balance), particularly in times of restricted physical activity due to pandemics.

**Objective:**

The objective of this study was to examine the effects of home-based balance exercises conducted during daily tooth brushing on measures of balance and muscle strength in healthy older adults.

**Methods:**

Fifty-one older adults were randomly assigned to a balance exercise group (*n* = 27; age: 65.1 ± 1.1 years) or a passive control group (*n* = 24; age: 66.2 ± 3.3 years). The intervention group conducted balance exercises over a period of eight weeks twice daily for three minutes each during their daily tooth brushing routine. Pre- and post-intervention, tests were included for the assessment of static steady-state balance (i.e., Romberg test), dynamic steady-state balance (i.e., 10-m single and dual-task walk test using a cognitive and motor interference task), proactive balance (i.e., Timed-Up-and-Go Test [TUG], Functional-Reach-Test [FRT]), and muscle strength (i.e., Chair-Rise-Test [CRT]).

**Results:**

Irrespective of group, the statistical analysis revealed significant main effects for time (pre vs. post) for dual-task gait speed (*p* < .001, 1.12 ≤ *d* ≤ 2.65), TUG (*p* < .001, *d* = 1.17), FRT (*p* = .002, *d* = 0.92), and CRT (*p* = .002, *d* = 0.94) but not for single-task gait speed and for the Romberg-Test. No significant group × time interactions were found for any of the investigated variables.

**Conclusions:**

The applied lifestyle balance training program conducted twice daily during tooth brushing routines appears not to be sufficient in terms of exercise dosage and difficulty level to enhance balance and muscle strength in healthy adults aged 60–72 years. Consequently, structured balance training programs using higher exercise dosages and/or more difficult balance tasks are recommended for older adults to improve balance and muscle strength.

## Introduction

The World Health Organization (WHO) recommends that adults aged ≥ 65 years should be at least 150 min physically active per week at moderate-intensity, or at least 75 min of vigorous-intensity physical activity throughout the week, or an equivalent combination of moderate- and vigorous-intensity activity. In addition, the WHO recommends exercise that stimulates balance and prevents falls for mobility limited older adults on three or more days per week. Finally, muscle-strengthening activities should be conducted for the major muscle groups on two or more days a week [1].

Unfortunately, there is evidence that 26 % of men and 35 % of women are insufficiently physically active in high-income countries, as compared to 12 % of men and 24 % of women in low-income countries [1]. Moreover, prevalence rates for insufficient physical activity increase with age from 30.1 % in adults aged 30–44 years to 46.1 % in older adults aged ≥ 60 years [2]. Research indicates that lifetime sedentary behavior may even accelerate biological aging of the neural, muscular, and skeletal systems which in combination cause high prevalence rates of dynapenia, sarcopenia, balance disorders, mobility limitations, and falls in older adults [3–6]. In a six year prospective cohort study, Heiland and colleagues [5] examined associations between balance and walking disorders and incident disability among adults with an initial mean age of 74 years. Disability was defined as the inability to complete one or more activities of daily living (ADL) such as eating, bathing, dressing, walking or moving around. Baseline balance (i.e., single leg stance time < 5 s) and walking speed limitations (preferred gait speed < 0.8 m/s) were both significantly associated with an increasing likelihood of subsequent development of ADL disability, even after controlling for the baseline number of chronic diseases and cognitive function. Consequently, interventions that promote balance and gait in older adults may have a positive effect on successful aging.

There is evidence from original research, systematic reviews, and meta-analyses that particularly interventions which include balance and strength exercises are effective in promoting mobility in older people [7–9]. For instance, Lacroix and colleagues showed that twelve weeks (3 sessions/week, 45 min per session) of combined and supervised balance and strength training significantly improved performance in measures of static (i.e., Romberg Test) and dynamic balance (i.e., stride velocity, Timed-Up-and-Go Test [TUG]) and muscle strength (i.e., Chair Rise Test [CRT]) in healthy older adults aged 73 years. In their systematic review with meta-analysis, Lesinski et al. [7] aggregated results from 23 balance training studies with healthy community-dwelling adults aged ≥ 65 years and found small to large sized effects for measures of static and dynamic balance. Dose-response relations for key balance training modalities were additionally extracted and showed that a training period of 11–12 weeks, a frequency of three sessions per week, a total number of 36–40 training sessions, a duration of 31–45 min of a single training session, and a total duration of 91–120 min of weekly balance training are particularly effective to improve older adults’ balance performance [7]. These study findings are based on supervised and group-based balance training programs.

During the COVID-19 pandemic, older adults who are at a disproportionately high risk to viral infections are restricted to their homes to perform physical exercise. Accordingly, home-based physical exercise programs constitute a feasible strategy to reduce the inactivity-induced mobility losses in older adults [10]. Hafström and colleagues [11] examined the effects of a six weeks home-based balance training program conducted four times per week for 16 min each in community-dwelling adults aged 60–80 years. After training, the intervention group showed improved one-legged standing by 32 % for eyes opened condition and 206 % for eyes closed condition. The grey literature reported that balance exercises should be included into daily tooth brushing routines to improve older adults’ balance performance, irrespective of the rather short exercise duration [12, 13]. This type of lifestyle exercise might be particularly suitable for sedentary older adults because the threshold to take up exercise is low. However, from a scientific point of view such statements need to be verified before being included in evidence-based recommendations.

In contrast to the 2010 WHO physical activity guidelines, the 2020 version [1] emphasizes that any duration of exercise even less than 10 min has the potential to induce health benefits. In addition, if the applied exercise stimulus is highly specific, chances may even increase to elucidate training effects [14]. Taken together, if balance exercises are conducted twice daily for three minutes each, the cumulated exercise volume might even be sufficiently high to induce specific effects in older adults’ balance performance. To the authors’ knowledge, this has not yet been studied which is why it appears timely to verify these reports.

Therefore, the aim of this study was to examine the effects of an eight week home-based balance exercise program conducted during daily tooth brushing on measures of static and dynamic balance and muscle strength in healthy older adults. In accordance with the principle of training specificity [15, 16], we hypothesized that balance exercises conducted during daily tooth brushing are effective in improving static balance with diminished transfer to dynamic and proactive balance as well as muscle strength.

## Methods

### Participants

Participants were recruited through flyers posted at public institutions (e.g., medical centers) and by advertisements in the local newspaper. Fifty-one community-dwelling healthy older adults (24 men, 27 women) between the ages of 60 and 72 years provided their written informed consent to participate in this study after experimental procedures were explained. The participants’ baseline characteristics are presented in Table 1. None of the participants had any history of diagnosed neurological (e.g., Parkinson disease) or orthopedic (e.g., joint replacement) disorders that might have affected their ability to perform home-based balance exercises conducted during daily tooth brushing or to perform tests for the assessment of balance and muscle strength. The participants were capable of walking independently without any assistive device and they had no prior experience with the applied tests or exercises. Participants were randomly assigned to an intervention (INT) or a passive control group (CON).

**Table 1:** Group-specific characteristics of the study participants (*N* = 51)

Characteristic	INT (*n* = 27)	CON (*n* = 24)	*p*-value
Age (years)	65.1 (1.1)	66.2 (3.3)	0.243
Sex (f/m)	15/12	12/12	
Body height (m)	1.69 (0.09)	1.71 (0.09)	0.600
Body mass (kg)	73.3 (13.0)	76.3 (11.6)	0.380
BMI (kg/m^2^)	25.2 (4.3)	26.5 (2.5)	0.552
CDT	all participants were classified as non-pathological		
MMSE (points)	28.5 (1.4)	28.3 (0.9)	0.192
FQoPA (h/week)	12.0 (10.5)	7.5 (5.4)	0.064

Notes: Values are means and standard deviations in parenthesis. *INT* intervention group (i.e., tooth brushing in combination with balance exercises); *CON* passive control group (i.e., tooth brushing only); *BMI* body mass index; *CDT *Clock Drawing Test; *MMSE* Mini-Mental State Examination; *FQoPA* Freiburg questionnaire of physical activity; *f* female; *m* male

### Experimental procedure

Upon entering the laboratory, participants were asked to fill out a number of questionnaires such as the Mini-Mental State Examination Test (MMSE), the Freiburg questionnaire of physical activity (FQoPA), and the Clock Drawing Test (CDT). The CDT is a sensitive screening test for the evaluation of executive function [17]. The elderly participants were asked to include the numbers of a clock in a given circle to make the circle look like a clock. Thereafter, participants were asked to draw the hands of the clock to a self-selected point in time. The next task was to translate the selected time in digital letters and to document those on the sheet. Depending on the study consulted, inter-rater reliability for the CDT ranges between 75.4 and 99.6 % [17]. Test-retest reliability can be classified as high, with a r-value of 0.90 [18]. Cross-correlation with the MMSE revealed a correlation coefficient of *r* > .50 [19]. As a result, the test distinguishes between pathological and normal test performance. The MMSE is a valid test of cognitive function. It separates patients with cognitive disturbance from those without such disturbance. Test-retest reliability for the MMSE is high, with *r* = .89. Cross-correlation with the Wechsler Adult Intelligence Scale score revealed a correlation coefficient of *r* = .78 [20]. An MMSE total score of < 24 separates patients with dementia or functional psychosis from cognitively independently functioning participants and those with anxiety neurosis or personality disorder. The FQoPA assesses the basic physical activity level (e.g., gardening, climbing stairs), leisure time physical activity (e.g., dancing, bowling), and sport activities (e.g., jogging, swimming) of people aged 18 to 78 years [21]. Age-specific corresponding norm values for total physical activity range between 9.9 and 13.6 h per week [21]. Significant test-retest reliability was reported for the summed physical activity level (*r* = .56). Data from the scientific literature indicated a significant cross-correlation (*r* = .42) with maximum oxygen uptake [21].

Prior to testing, all participants received standardized verbal instructions regarding test procedures with a visual demonstration of the balance and muscle strength tests. Thereafter, participants performed a 5-minutes warm up consisting of bipedal and monopedal balance exercises as well as submaximal stepping and skipping movements. Pre- and post-intervention, tests were conducted for the assessment of static steady-state balance (i.e., Romberg test), dynamic steady-state balance (i.e., 10-m single and dual-task walk test), proactive balance (i.e., TUG, Functional-Reach-Test [FRT]), and muscle strength (i.e., CRT). Balance tests were always conducted in randomized order and prior to the muscle strength test. This test sequence was applied in order to keep the effects of neuromuscular fatigue minimal. All participants received one familiarization trial for each test. Thereafter, one test trial was conducted unless otherwise stated.

### Balance exercises while tooth brushing

Participants of the INT-group conducted a lifestyle exercise program which included balance exercises conducted during the daily tooth brushing routine. According to Creeth et al. [22], the teeth were brushed twice per day (i.e., in the morning, right after getting up and at night, before going to bed) for three minutes each on seven days per week for a duration of eight weeks resulting in a total of 112 exercise sessions. This equals an overall exercise time of 336 min and a weekly exercise time of 42 min. The exercise protocol is illustrated in detail in Table 2. Before the exercise period started, participants of the INT-group received information on how to perform the balance exercises by the authors of this article. Additionally, exercise cards containing pictures and descriptions on how to correctly perform all exercises were provided for the eight weeks exercise period. Moreover, participants of the INT-group were asked during weekly phone calls whether they need more information on how to properly perform the balance exercises. By doing so, we wanted to make sure that the exercises were performed with adequate movement skill competence. All participants had to document the realized exercise sessions in a training log. The balance enhancing exercises were performed barefooted or alternatively with socks under different stance and surface conditions. Progression during the balance exercise program was achieved by continuously reducing the base of support (i.e., from step over tandem to one-legged stance) and by including an unstable surface (i.e., rolled up towel). This exercise sequence for progression in balance training has been validated in a previously published study [23]. The participants of the passive CON-group did not receive any intervention during the study period.

**Table 2:** Protocol of the eight weeks balance exercise program for the intervention group conducted twice daily during tooth brushing routines

Balance exercise protocol	
Exercises	• Step stance (week 1: on stable surface [i.e., floor], week 2: on unstable surface [i.e., rolled up towel]) • Tandem stance (week 3: on stable surface, week 4: on unstable surface [i.e., rolled up towel]) • One-legged stance^a^ (weeks 5 & 6: on stable surface, weeks 7 & 8: on unstable surface [i.e., rolled up towel])
Training load	• 8 weeks • 7 days per week • 2 sessions per day (i.e., in the morning and the evening) • 3 min per session
Progression during training	• Continuous reduction in base of support (i.e., from step over tandem to one-legged stance) and inclusion of an unstable element (i.e., rolled up towel)

^a^Participants were instructed to use the dominant leg first and to switch to the non-dominant leg as they felt safe and comfortable to do so. Given that participants concurrently brushed their teeth and performed balance exercises, the applied exercise program can be considered a dual-task program including a primary balance task and a secondary motor interference task (i.e., brushing teeth)

### Assessment of static, dynamic, and proactive balance

Test circumstances (e.g., room illumination, temperature, noise) during balance assessment were in accordance with recommendations for posturographic testing [24]. Static steady-state balance was assessed using the Romberg Test [24]. Participants performed four tasks with an increasing level of difficulty: (1) standing in an upright position with feet closed and eyes opened for 10 s without swaying while holding both arms extended in horizontal direction with palms facing upwards; (2) ditto, but with eyes closed; (3) ditto, but eyes opened and feet in tandem stance; (4) ditto, but eyes closed and feet in tandem stance. Standing time during the different test conditions was recorded using a stopwatch to the nearest 0.1 s. Maximal stance time for the fourth task was used for further analysis. Age-specific corresponding norm values are 14.0 to 15.0 s for females and 14.3 to 17.5 s for males [24]. High test-retest reliability has been shown for the Romberg Test (eyes opened, intraclass correlation coefficient [ICC] = 0.86 and eyes closed, ICC = 0.84) and Sharpened Romberg Test (eyes opened, ICC = 0.70 and eyes closed, ICC = 0.91) [25].

Dynamic steady-state balance was assessed using the 10-m walking test. Participants walked with their own footwear at self-selected speeds, initiating and terminating each walk a minimum of one meter before and after the 10-m walkway to allow sufficient distance to accelerate to and decelerate from a steady state of ambulation across the walkway. Time was recorded with a stopwatch to the nearest 0.1 s. Gait speed (m/s) was determined from the time needed to cover the 10-m walking distance under single-task (walking only) and dual-task conditions (walking while concurrently performing a cognitive interference task or a motor interference task). Age-specific norm values for single-task gait speed are 1.10 m/s for females and 1.12 m/s for males [26].

Proactive balance was assessed using the TUG and the FRT. The TUG was used as described by Podsiadlo and Richardson [27]. Participants were asked to perform the TUG at their self-selected habitual walking speed. Time was recorded with a stopwatch to the nearest 0.1 s. Participants were seated and instructed to walk three meters, turn around, walk back to the chair and sit down. The stopwatch was started on the command “ready-set-go” and stopped as the participant sat down. Age-specific corresponding norm values are 8.0 to 9.0 s for both sexes [28]. The TUG showed excellent test-retest reliability (ICC = 0.99) in older adults [27]. Proactive balance was further assessed by means of the FRT. The FRT measures the maximal distance one can reach forward beyond arm’s length while maintaining a fixed base of support in the standing position [29]. Maximal reach distance of the right arm was recorded to the nearest 0.5 cm. Age-specific norm values are 29.0 to 30.0 cm for both sexes [30]. The FRT showed excellent test-retest reliability (ICC = 0.92) in older adults [29]. Validity of the FRT has previously been shown by Newton et al. [31] when testing healthy community-dwelling older adults.

### Assessment of muscle strength

The CRT as described by Csuka and McMarty [32] was used for the assessment of lower limbs muscle strength. More precisely, participants sat on a chair with their arms crossed in front of their chest. On the command ready, set, go, participants stood up and sat down as quickly as possible for five times. Three test trials were performed which were separated by a 1-minute rest interval and the best (least time) out of three trials was used for further analysis. Time was recorded with a stopwatch to the nearest 0.1 s. Age-specific norm values have been reported for females (12.7–13.0 s) and males (8.4–11.6 s) [33]. High test-retest reliability has previously been shown for the CRT (ICC = 0.89) [34].

### Cognitive and motor interference tasks

Dynamic steady-state balance (i.e., 10-m walking test) was also examined while performing a concurrent attention-demanding cognitive or motor interference task. The cognitive interference task comprised an arithmetic task in which the participants loudly recited serial subtractions by three, starting from a randomly selected number between 300 and 900 given by the experimenter [35]. The motor interference task required participants to hold two interlocked sticks steadily in front of their body. One stick was held in each hand, with the elbow in 90-degree flexion. Each stick had a ring at the end with a diameter of four cm, and the rings were interlocked [36]. The participants were advised not to let the rings touch each other. When the dual-task methodology was used, participants were instructed to give equal priority to both tasks in order to create real-life conditions [37].

### Statistical analyses

Descriptive data are presented as group mean values and standard deviations (SD). After normal distribution was examined and confirmed using the Kolmogorov-Smirnov-Test, an independent samples *t*-Test was applied to determine significant between group baseline differences. Subsequently, a 2 (groups: INT, CON) × 2 (time: pre, post) analysis of variance (ANOVA) with repeated measures on time was used. The classification of effect sizes was determined by converting partial eta-squared to Cohen’s *d*. The effect size is a measure of the effectiveness of a treatment and it helps to determine whether a statistically significant difference is a difference of practical concern. According to Cohen [38], effect sizes can be classified as small (0 ≤ *d* ≤ 0.49), medium (0.50 ≤ *d* ≤ 0.79), and large (*d* ≥ 0.80). The significance level was set at *p* < .05. An a priori power analysis [39] with an assumed type I error rate of 0.05 and a type II error rate of 0.20 (80 % statistical power) was conducted for balance measures [40] and revealed that 25 participants per group would be sufficient for revealing interaction effects. Due to potential drop-outs, a total of 55 older adults were enrolled in this study, 29 in INT and 26 in CON. All analyses were performed using the Statistical Package for Social Sciences (SPSS) version 26.0.

## Results

Findings from the CDT, MMSE, and FQoPA imply that our study participants were cognitively healthy and physically active (Table 1). All participants of the INT-group received the balance training program as allocated. During the intervention period, two INT and two CON individuals dropped-out due to personal reasons. Thus, 27 participants (INT-group) finally completed the home-based exercise program and none reported any training or test-related injury. Adherence rate (i.e., percentage rate of the conducted overall exercise sessions) for the INT-group was 92 % (i.e., tooth brushing in combination with balance exercises) and 91 % for CON-group (i.e., tooth brushing only). Table 3 displays means and SDs for all analyzed variables. No statistically significant baseline between group differences were detected for all parameters.

**Table 3:** Effects of an eight weeks home-based balance exercise program conducted during tooth brushing on measures of balance and muscle strength in healthy older adults

	INT (*n* = 27)			CON (*n* = 24)			*p*-value (Cohen’s *d*)		
Variables	Pre	Post	Δ [%]*	Pre	Post	Δ [%]*	Time	Group	Group × Time
*Balance*									
Romberg-Test (s)	24.2 (9.2)	25.2 (9.1)	+ 4	21.6 (10.7)	23.3 (10.3)	+ 8	0.22 (0.36)	0.38 (0.26)	0.74 (0.09)
Gait speed, ST (m/s)	1.4 (0.2)	1.5 (0.2)	+ 3	1.4 (0.2)	1.4 (0.1)	-1	0.46 (0.21)	0.41 (0.24)	0.30 (0.30)
Gait speed, DT-CI (m/s)	1.2 (0.4)	1.3 (0.3)	+ 15	1.2 (0.2)	1.3 (0.2)	+ 9	< 0.001 (1.12)	0.66 (0.13)	0.37 (0.26)
Gait speed, DT-MI (m/s)	1.1 (0.3)	1.4 (0.3)	+ 29	1.1 (0.2)	1.3 (0.2)	+ 22	< 0.001 (2.65)	0.40 (0.24)	0.21 (0.36)
TUG (s)	8.3 (1.3)	7.7 (1.2)	+ 7	8.3 (1.2)	8.0 (1.3)	+ 4	< 0.001 (1.17)	0.63 (0.14)	0.11 (0.47)
FRT (cm)	36.2 (5.3)	38.2 (4.7)	+ 6	35.2 (6.6)	38.8 (5.8)	+ 10	0.002 (0.92)	0.90 (< 0.01)	0.34 (0.28)
*Muscle strength*									
Chair rise test (s)	10.1 (2.8)	8.2 (2.1)	+ 19	9.6 (1.7)	9.0 (1.6)	+ 6	0.002 (0.94)	0.77 (0.09)	0.11 (0.47)

Notes: Vales are means and standard deviations in parenthesis. *A positive/negative percentage value indicates a performance improvement/decrement. *d* effect size; *CI* cognitive interference; *DT* dual-task; *MI* motor interference; *FRT* Functional Reach Test; *ST* single-task; *TUG* Timed Up and Go Test

### Static, dynamic, and proactive balance

For the Romberg-Test and gait speed during single-task walking, neither main effects for time and group nor a significant group × time interaction was found (Table 3). During dual-task conditions, significant main effects for time were found for cognitive interference walking (*F*_1, 49_ = 15.32, *p* < .001, *d* = 1.12) and motor interference walking (*F*_1, 49_ = 86.04, *p* < .001, *d* = 2.65) (Table 3). For both parameters, no significant main effects for group or group × time interactions were observed. In addition, significant main effects for time were observed for the TUG (*F*_1, 49_ = 16.69, *p* < .001, *d* = 1.17) and the FRT (*F*_1, 49_ = 10.41, *p* = .002, *d* = 0.92) (Table 3). However, we could not find any significant main effects for group nor group x time interactions.

### Muscle strength

For CRT, a significant main effect for time was detected (*F*_1, 49_ = 10.90, *p* = .002, *d* = 0.94). The main effect for group and the group x time interaction did not reach the level of significance (Table 3).

## Discussion

To the authors’ knowledge, this is the first study that investigated the effects of an eight week home-based balance exercise program conducted twice daily during tooth brushing routines on various measures of balance and muscle strength in cognitively healthy and physically active adults aged 60 to 72 years. The main findings of this study were that (i) the performance of balance exercises while tooth brushing appears to be feasible (i.e., attendance rate: 92 %) and safe (i.e., no training-related injuries) for healthy older adults; (ii) dynamic steady-state balance (i.e., gait speed while concurrently performing a cognitive or motor interference task), proactive balance (i.e., TUG, FRT), and muscle strength (i.e., CRT) significantly improved in both experimental groups following eight weeks; (iii) static steady-state balance (i.e., Romberg-Test) and dynamic steady-state balance (i.e., single-task gait speed) did not significantly change; (iv) no significant group-by- time interactions were found for any of the investigated variables. In light of these results, our initial hypothesis stating that balance exercises conducted during daily tooth brushing are effective in improving static balance with diminished transfer to dynamic and proactive balance and muscle strength was not confirmed.

There is evidence in the literature that unipedal stance time is associated with a history of falling in older adults [41]. In fact, outpatients with a mean age of 66 years and a history of falls showed significantly shorter unipedal stance times (9.6 ± 11.6 s) compared with individuals who had not fallen (31.3 ± 16.3 s). These authors [41] concluded that an unipedal stance time < 30 s in an older ambulatory outpatient population is associated with a history of falling, while an unipedal stance time ≥ 30 s is associated with a low risk of falling [41]. Moreover, Heiland and colleagues [5] were able to show that baseline balance (i.e., unipedal stance time < 5 s) and walking speed limitations (preferred gait speed < 0.8 m/s) were both significantly associated with an increasing likelihood of subsequent development of ADL disability, even after controlling for the baseline number of chronic diseases and cognitive function. Accordingly, exercise-induced improvements in unipedal stance time may have a positive impact on seniors’ mobility and fall risk. We hypothesized that home-based balance exercises conducted during daily tooth brushing are effective in improving static balance with diminished transfer to dynamic and proactive balance and muscle strength. This easy-to-administer and time efficient exercise program appears to be of high relevance during the COVID-19 pandemic in which older adults are at a disproportionately high risk to viral infections and therefore restricted to their homes to perform physical exercise. While reports from the grey literature [12, 13] imply positive effects of balance exercises conducted during daily tooth brushing, experimental data from our study showed that this exercise stimulus was insufficient to improve measures of static steady-state balance (i.e., Romberg-Test) and dynamic steady-state balance (i.e., single-task gait speed). Participants from the INT-group brushed their teeth twice per day (i.e., in the morning, right after getting up and at night, before going to bed) for three minutes each on seven days per week for a duration of eight weeks resulting in a total of 112 exercise sessions. This equals an overall exercise time of 336 min and a weekly exercise time of 42 min. The applied weekly exercise dosage is admittedly not in accordance with the WHO recommendations for adults ≥ 65 years [1]. However, the 2020 WHO guidelines [1] emphasize that any duration of exercise even less than 10 min has the potential to induce health benefits. In addition, the applied training program was progressively designed over the course of the intervention and the exercise stimulus was highly specific and therefore in agreement with the principle of training specificity which is why we expected exercise-induced improvements despite the rather low weekly exercise dosage. In addition to our findings on static steady-state balance (i.e., Romberg-Test) and dynamic steady-state balance (i.e., single-task gait speed), we detected significant improvements in measures of dynamic steady-state balance (i.e., gait speed while concurrently performing a cognitive or motor interference task), proactive balance (i.e., TUG, FRT), and muscle strength (i.e., CRT). However, the enhancements were observed in both experimental groups which is why they cannot be solely attributed to the applied balance exercise program.

Three reasons may account for the missing or not clearly assignable intervention effects in this study. First, we do not have any objective proof that verifies the high mean adherence rate (92 %) reported by the individuals participating in the INT-group. Prior to the start of the study, participants were asked to complete a training log on a daily basis and they received weekly phone calls to increase the likelihood that they really performed the exercises. Despite these means, we cannot rule out that the reported adherence rate overestimates true adherence to training. Second, before the study started, all participants of the INT-group received information on how to perform the balance challenging exercises by the authors of this article. Additionally, exercise cards containing pictures and descriptions of the respective exercises were provided for the eight weeks exercise period. Even though these information were delivered to all INT-individuals, training was unsupervised and movement skill competence during the performance of the exercises may not have been sufficient. In addition, it has to be noted that INT-participants performed a specific type of dual-task exercise program. In other words, while doing their balance exercises they concurrently brushed their teeth. This could have caused interference and may have resulted in diminished performance in one or both concurrently conducted single tasks (balance exercise and/or tooth brushing). Given that the enrolled participants were cognitively healthy and physically active, we think however that they should have been capable of performing this specific type of dual-task balance training.

Having said this, the third and most likely reason for the observed non-significant interaction findings is that the exercise dosage was too low and/or the initial fitness level of the participants too high. Lesinski et al. [7] aggregated dose-response relations for key balance training modalities and found that a total duration of 91–120 min of weekly balance training was particularly effective to improve older adults’ balance performance. The applied weekly exercise dosage in this study was 42 min, which is less than half of the recommended balance dosage. Therefore, older adults are advised to follow the balance training guidelines provided by Lesinski and colleagues [7]. Table 3 illustrates participants’ baseline balance and strength levels. An important marker of older adults’ mobility and health status is walking speed which is why it has previously been denoted as the sixth vital sense [42]. The participants of the INT- and CON-group achieved an average baseline gait speed of 1.45 m/s and 1.44 m/s, respectively. In their review article, Abellan van Kan et al. [43] provided cut-points of gait speed at usual pace for older adults. With reference to the study of Studenski et al. [43], they classified adults ≥ 65 years with a gait speed > 1.3 m/s as extremely fit. When taking this baseline fitness level of our participants into account, we may also have experienced ceiling effects. Besides exercise dosage and progression, exercise selection constitutes another important programming parameter that should be considered for individualized exercise prescription. In this study, balance training progression was most likely adequate to induce adaptive processes because it was in line with an established sequence for progression in balance training. However, exercise dosage may have been too low, especially when considering the high baseline fitness levels of our subjects [23]. Finally, the selection of balance exercises may have been too monotonous and thus not challenging enough to sufficiently stimulate the postural control system. Basically, the applied program consisted of three static balance exercises performed during step stance, tandem stance, and one-legged stance. These three key balance exercises may not have been sufficiently challenging to continuously stimulate the sensorimotor system over the eight weeks study period [11].

There are a number of limitations related to this study that warrant discussion. Although, we induced progression over the course of the eight weeks balance exercise program by systematically reducing the base of support (i.e., from step over tandem to one-legged stance) and by including an unstable surface (i.e., rolled up towel), we cannot completely rule out that training intensity was still too low. The regulation of the program parameter ‘intensity’ is more difficult for balance compared with strength training. In other words, more research is needed to find appropriate ways on how to apply sufficient training intensity during the regular performance of balance exercises. The findings of this study are specific to the population under investigation. Hypothetically, mobility limited older adults or individuals with balance deficits may particularly benefit from such a program. However, from an ethical point of view, it appears problematic if mobility limited older adults perform a home-based unsupervised exercise program. Finally, the application of tests using biomechanical apparatus (e.g., force plates) instead of clinical tests could have increased test sensitivity and therefore chances to find intervention effects. A review paper [44] summarized sensitivity and specificity of the applied clinical tests and we consider those acceptable.

## Conclusions

The results of this study illustrate that balance training during daily tooth brushing is feasible and safe for healthy older adults. Given that both tasks were concurrently performed but did not cause harm to the participants, we propose this exercise combination as time efficient and easy-to-administer if the goal is to maintain the already existing level of postural control. This type of lifestyle exercise program might be particularly appealing for sedentary older adults without mobility limitations because the threshold to take up exercise is low.

## Data Availability

The datasets used and/or analyzed during the current study are available from the corresponding author on reasonable request.

## Abbreviations

ADL: Activities of daily living
ANOVA: Analysis of variance
CDT: Clock Drawing Test
CI: Cognitive interference task
CON: Control
CRT: Chair Rise Test
DT: Dual-task
FRT: Functional-Reach-Test
FQoPA: Freiburg questionnaire of physical activity
ICC: Intraclass correlation coefficient
INT: Intervention
MI: Motor interference task
MMSE: Mini-Mental State Examination Test
SD: Standard deviation
ST: Single-task
TUG: Timed-Up-and-Go Test
WHO: World Health Organization
